# On-table monitoring of prostate MRI could enable tailored utilisation of gadolinium contrast

**DOI:** 10.1007/s00330-025-11479-3

**Published:** 2025-03-15

**Authors:** Tom Syer, Bruno Carmo, Nimalam Sanmugalingam, Brooke Lawson, Wellington Chishaya, Christopher Shepherd, Tristan Barrett, Iztok Caglic

**Affiliations:** 1 Department of Radiology, Addenbrookes Hospital, Cambridge University Hospitals NHS Foundation Trust, Cambridge, UK; 2 Department of Radiology, University of Cambridge, Cambridge, UK

**Keywords:** Prostatic neoplasms, Magnetic resonance imaging, Contrast media

## Abstract

**Objectives:**

To compare the impact of on-table monitoring vs standard-of-care multiparametric MRI (mpMRI) for the utilisation of gadolinium contrast use in prostate MRI.

**Materials and methods:**

This retrospective observation study of prospectively acquired data was conducted at a single institution over an 18-month period. A cohort of patients undergoing MRI for suspected prostate cancer (PCa) underwent on-table monitoring where their T2 and DWI images were reviewed by a supervising radiologist during the scan to decide whether to acquire dynamic contrast-enhanced (DCE) sequences. MRI scans were reported using PI-RADS v2.1, patients were followed up with biopsy for at least 12 months. The rate of gadolinium administration, biopsy rates, and diagnostic accuracy were compared to that of a standard-of-care control group undergoing mpMRI during the same period using propensity score matching. Estimates of cost savings were also calculated.

**Results:**

1410 patients were identified and after propensity score matching 598 patients were analysed, with 178 undergoing on-table monitoring. Seventy-five and eight tenths (135/178) of patients did not receive gadolinium. Contrast was used mainly for indeterminate lesions (27/43) and significant artefacts on bpMRI (14/43). When comparing the monitored cohort to a non-monitored control group, there was a comparable number of biopsies performed (52.2% vs 49.5%, *p* = 0.54), PI-RADS 3/5 scoring rates (10.1% vs 7.4%, *p* = 0.27), sensitivity (98.3% vs 99.2%, *p* = 0.56), and specificity (63.9% vs 70.7%, *p* = 0.18) for detection of clinically-significant PCa. When acquired, DCE was deemed helpful in 67.4% (29/43) of cases and improved both PI-QUALv2 and reader confidence scores. There was an estimated saving of £56,677 over the 18-month study.

**Conclusion:**

On-table monitoring significantly reduced the need for gadolinium contrast without compromising diagnostic accuracy and biopsy rates.

**Key Points:**

***Question*** *Default use of gadolinium contrast in prostate MRI is not always of clinical benefit and has associated side effects and healthcare costs*.

***Findings*** *On-table monitoring avoided the use of gadolinium in 75.8% of patients, reducing associated costs whilst maintaining clinically significant cancer detection, and diagnostic accuracy and improving reader confidence*.

***Clinical relevance*** *On-table monitoring offers personalised patient protocolling with a significant reduction in the use of gadolinium and its associated side effects and costs, potentially maximising the advantages of both multiparametric and biparametric prostate MRI*.

**Graphical Abstract:**

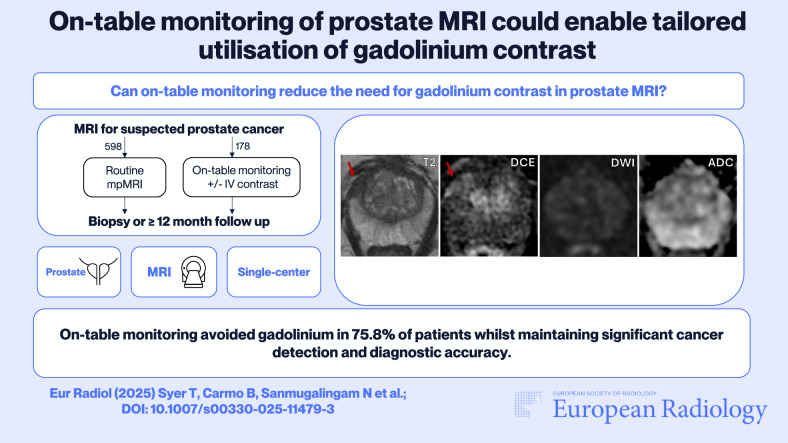

## Introduction

Magnetic resonance imaging (MRI) is now well-established for the initial diagnosis of suspected localised prostate cancer (PCa). Current international guidelines, recommend multiparametric (mp) MRI be performed according to the prostate imaging-reporting and data system v2.1 (PI-RADSv2.1) standards, including the three key sequences of T2-weighted (T2WI), diffusion-weighted (DWI), and dynamic contrast-enhanced (DCE) imaging [1–4]. DCE sequences require intravenous injection of a gadolinium-based contrast and are considered positive when a suspicious area demonstrates focal early enhancement earlier than or contemporaneously with adjacent background tissue.

It has been proposed that DCE may not be required for all cases either in the initial diagnostic setting or for monitoring during active surveillance [5, 6]. DCE plays a less dominant role in the PI-RADSv2.1 scoring system, used only to upgrade indeterminate peripheral zone lesions [3], leading to a limited additional diagnostic benefit over T2 and DWI [7]. There are potential risks from contrast agents, including allergic reactions, nephrogenic systemic fibrosis in patients with renal failure and intracranial deposition of gadolinium [8, 9]. Biparametric MRI (bpMRI) omitting DCE benefits from reduced costs, faster scanning time, and increased flexibility for scheduling due to lower requirements for medical supervision, which may help widen access to pre-biopsy MRI [5, 10–12].

There is growing retrospective evidence to suggest the equal effectiveness of bpMRI vs mpMRI [13–16]. BpMRI without DCE, however, may result in higher levels of uncertainty by increasing the rate of indeterminate PI-RADS 3 scores [13, 14], DCE can improve overall sensitivity for detecting clinically significant tumours, and be a helpful “safety net”, particularly for less experienced readers and complex cases [15, 16]. In addition, when there are significant artefacts on other sequences from motion, pelvic metallic implants or rectal gas, DCE may aid in diagnosis [3]. For these reasons, the default use of bpMRI necessitates high-quality imaging and expert readers and some patients may always benefit from DCE [3, 11, 17, 18].

Predicting these patients prospectively may be difficult, and although patients could be recalled to the department for additional DCE sequences, this process is inefficient [13–16]. One option suggested by the PI-RADS committee, is for on-table monitoring of patients following the acquisition of the T2WI and DWI sequences, with a subsequent decision on the need for contrast injection for select patients [11]. This approach would maintain the overall advantages of a bpMRI approach, whilst also enabling contrast administration to patients who would most benefit from undergoing mpMRI. In this study, we prospectively compared the use of on-table monitoring to the standard approach of mpMRI for all patients and its impact on the utilisation of gadolinium contrast and its effect on MRI reporting, biopsy rates and cancer detection.

## Materials and methods

This study was a retrospective review of prospectively acquired data at a single institution and was approved as part of a prostate MRI service evaluation, with the need for informed consent for data analysis waived by the local ethics committee (CUH/018/PRN7919). The study population comprised consecutive biopsy naïve patients referred with a suspicion of PCa who underwent prostate MRI between November 2021 and May 2023. Patients were excluded if they had a previous diagnosis of PCa, treatment for benign or malignant prostatic disease or when the biopsy was not performed despite the high probability of significant disease due to other clinical factors.

### MRI examination

All images were acquired from one of three scanners from the same vendor (GE Healthcare) at both 1.5 T and 3 T field strengths. The protocol for all patients included axial T1-weighted imaging, axial and sagittal high-resolution T2-weighted imaging (T2WI), and axial diffusion-weighted imaging (DWI) with corresponding apparent diffusion coefficient (ADC) maps. When required, an axial DCE MRI was also acquired following the injection of gadobutrol (Gadovist, Bayer HealthCare). Full details of the MRI sequence parameters are presented in Supplementary Table 1.

### On-table monitoring and MRI reporting

During one reporting session per week over the 18-month period, patients were prospectively supervised by a radiologist during MRI scanning to decide on the use of intravenous contrast medium. The remaining patients imaged during this period underwent mpMRI without on-table monitoring. All MRI examinations were reported according to PI-RADS v2.1 standards, and patients were subsequently managed by local standard-of-care practices [3].

Supervision was done by one of two expert uroradiologists with 15 years’ (T.B.) and 10 years’ (I.C.) experience in prostate MRI reporting, and considered experts based on a number of mpMRIs reported [19, 20]. During monitored lists the radiologist would review the initially acquired T2 and DWI sequences whilst the patient was still in the scanner, then would decide if adding DCE sequences would be beneficial and record their reasoning, informing the MRI radiographer performing the study. If DCE images were felt to be of benefit, they would then be acquired. The radiologists also assigned a 1-5 Likert score for their confidence in making an assessment, as previously described [21], with just bpMRI sequences and again for mpMRI if DCE was acquired. Readers also commented on whether or not DCE helped their reporting and why. PI-QUALv2 scores were retrospectively assigned to both the bpMRI and mpMRI for patients within the on-table monitoring cohort who underwent DCE imaging [22].

### Reference standard and follow-up

All patients underwent either targeted MRI-US fusion biopsy or were followed up for a minimum of 12 months, with at least one repeat PSA test. The decision for biopsy was made by the shared decision-making between the urologist and the patient. Clinically significant PCa was defined as Gleason grade ≥ 3 + 4 on biopsy.

### Cost analysis

The NHS England National Tariff Payment System 2022/23 was referenced to estimate cost savings from a potential reduction in the use of gadolinium contrast. The tariff figures for a one-part MRI examination with and without contrast are £169 and £116, respectively [23].

### Statistical analysis

Due to the non-randomised nature of the on-table monitoring group, nearest neighbour propensity score matching with Mahalanobis-metric matching was conducted using a 1:3 matching ratio between the on-table and non-monitoring groups, respectively. Age, PSA, PSAd and prostate volume baseline covariates were used for matching. Subsequently, descriptive statistics were calculated for baseline characteristics between non-monitored and on-table monitoring cohorts. Mann–Whitney hypothesis testing was used for non-parametric continuous variables and Pearson’s Chi-squared test for categorical variables. The proportion of patients receiving contrast, and differences in the rates of biopsy, clinically significant and insignificant cancer detection and PI-RADS 3 scores were reported. Sensitivity, specificity, negative, and positive predictive values were calculated for each group using a PI-RADS v2.1 cut-off score ≥ 3. Wilson’s 95% confidence intervals were calculated where applicable and hypothesis testing using a two-sample test of proportions was performed. All *p*-values were two-sided, and values ≤ 0.05 were deemed significant. All statistical analysis was conducted using STATA version 17.0.

## Results

**Fig. 1:**
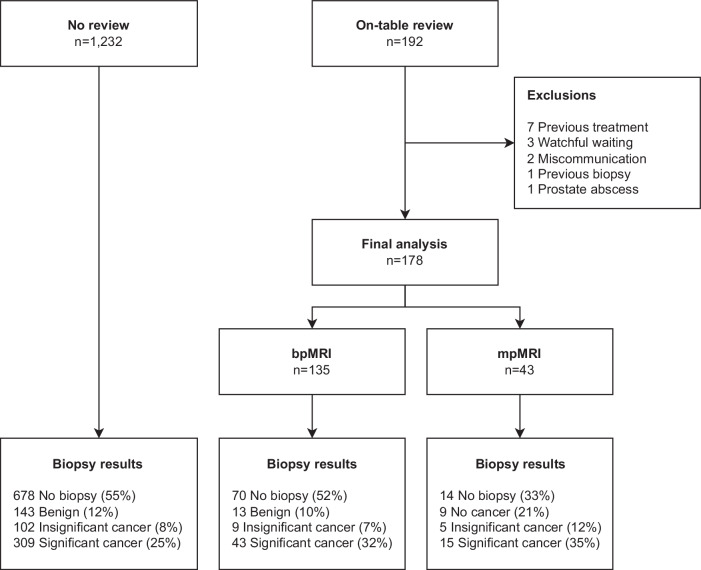
Participant flow chart and biopsy outcomes. bpMRI, biparametric MRI; mpMRI, multiparametric MRI

The final cohort after exclusions totalled 1410 patients with a median age of 67 years (IQR 61–73 years), PSA of 5.71 ng/mL (IQR 3.98–8.57 ng/mL), prostate volume of 56.0 mL (IQR 38.3–79.0 mL) and PSAd of 0.10 ng/mL^2^ (IQR 0.07–0.16 ng/mL^2^). From this cohort 178 underwent on-table monitoring, and following propensity score matching 420 patients were included in the non-monitoring comparison group. Fig. 1 shows a flow chart chart of the study cohort, exclusions and outcomes, and Table 1 presents the baseline characteristics between the two matched groups. Two patients were excluded from the on-table monitoring group due to miscommunication resulting in the acquisition of standard-of-care mpMRI imaging.

**Table 1:** Baseline demographics for the on-table monitoring and no-monitoring cohort

Cohort	On-table monitoring	No monitoring	*p*-value
	*n* = 178	*n* = 420	
Age, years	68 (62, 74)	68 (62, 74)	0.90
PSA, ng/mL	6.4 (4.3, 9.4)	6.0 (4.1, 8.4)	0.34
Prostate volume, mL	57.0 (38.0, 77.0)	57.0 (38.8, 78.8)	0.97
PSAd, ng/mL^2^	0.11 (0.07, 0.18)	0.10 (0.07, 0.16)	0.39

*PSA* prostate specific antigen, *PSAd* prostate specific antigen density

**Fig. 2:**
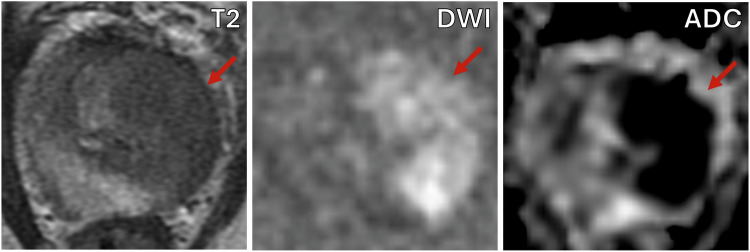
Seventy-year-old man, PSA 6.0. T2-weighted imaging (T2) shows a 28 mm lesion in the left PZ with corresponding restricted diffusion on DWI and ADC map. Dynamic contrast-enhancing imaging (DCE) would not change the decision for biopsy. Targeted biopsy revealed Gleason score 4 + 4 = 8 (Grade Group 4), max tumour length in a single core = 7 mm with extra-prostatic extension was seen

**Fig. 3:**
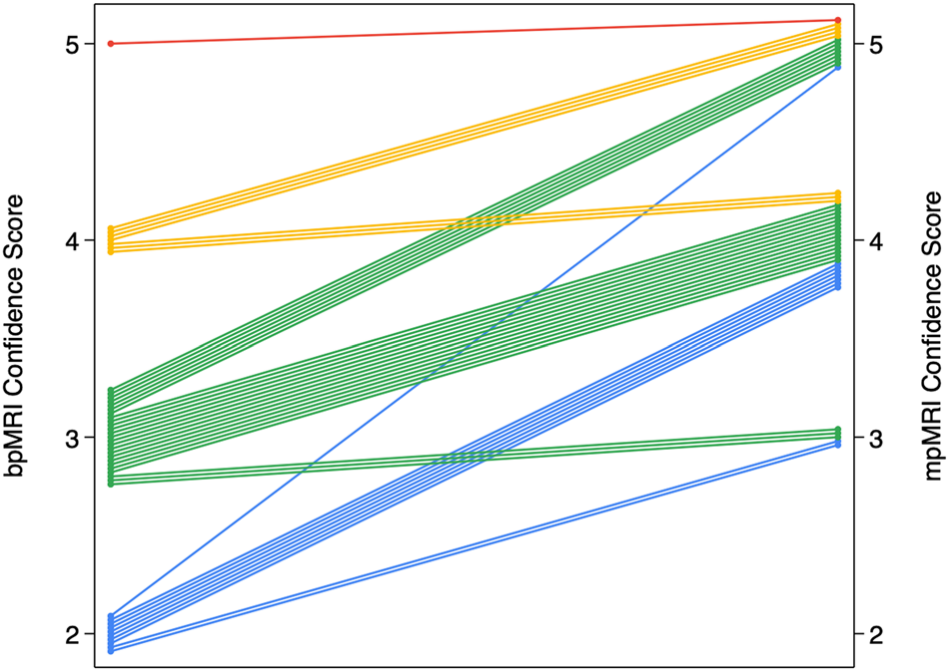
Change in confidence scores after the addition of DCE for patients undergoing mpMRI in the on-table monitoring group. The left-hand axis represents the confidence score for bpMRI before DCE use and the right-hand axis the confidence scores for mpMRI. The original bpMRI confidence scores of 2, 3, 4, and 5 are colour-coded as blue, green, yellow and red, respectively. The scores for the same individual patients before and after DCE are joined with a single straight-line

**Fig. 4:**
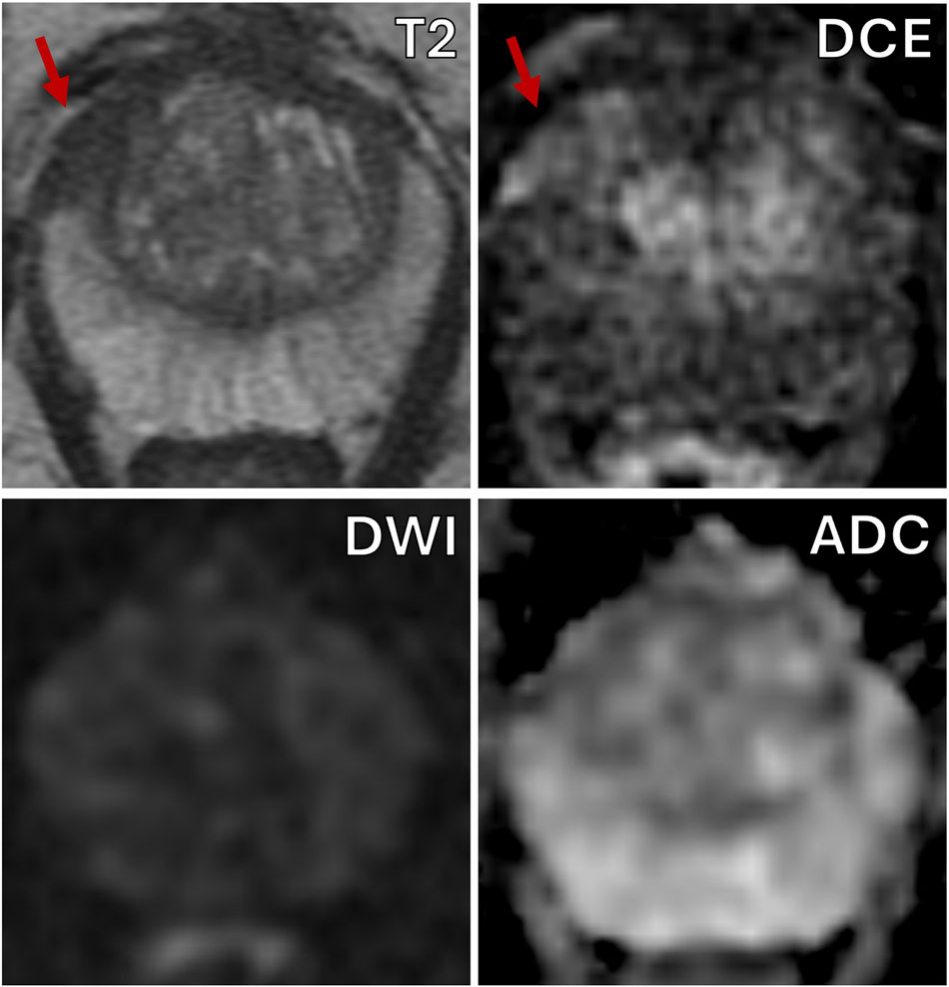
Sixty-year-old man, PSA 4.1. T2-weighted imaging (T2) shows a 12 mm lesion in the right anterior PZ (arrow) but with only subtle restricted diffusion on the DWI and ADC map. DCE imaging helps confirm corresponding focal enhancement (arrow). Targeted biopsy revealed Gleason score 3 + 4 = 7 (Grade Group 2), max tumour length in a single core = 7 mm

Within the on-table monitoring group, 135/178 (75.8%; 95% CI: 69.0, 81.6%) patients were not given intravenous contrast, the baseline characteristics between those receiving and not receiving contrast are presented in Table 2. The reasons for not giving contrast were: PI-RADS score 1–2 with no lesion identified (79/135), or a clear PI-RADS ≥ 4 lesion was seen on bpMRI (56/135), an example is shown in Fig. 2. The reasons for giving contrast were indeterminate lesions on bpMRI (27/43), the presence of artefact significantly affecting interpretation (14/43), and diffuse peripheral zone changes (3/43). When contrast was given, the radiologists felt it subjectively to be helpful in 29/43 patients (67.4%; 95% CI: 52.5, 79.5%) and the mean confidence score for interpretation increased from 3.0 to 4.2 (*p* < 0.001). Individual patient changes in confidence score with the addition of contrast are shown in Fig. 3. 13/27 PI-RADS 3 lesions showed focal enhancement and were up-scored to PI-RADS 4 (“3 + 1”), an example is shown in Fig. 4. The prevalence of significant cancer was similar between PI-RADS “3 + 1” and other PI-RADS 4 lesions based on bpMRI with 7/13 (53.8%; 95% CI: 29.1, 76.8%) compared to 15/30 (50.0%; 95% CI: 33.2, 66.8%) cases, respectively.

**Table 2:** Comparison between on-table monitoring cohort patients who underwent biparametric or multiparametric MRI

On-table review cohort	bpMRI	mpMRI	*p*-value
	*N* = 135	*N* = 43	
Age, years	68 (63, 74)	65 (58, 73)	0.18
PSA, ng/mL	6.7 (4.5, 10.3)	5.4 (3.7, 8.0)	0.04
Prostate volume, mL	60.4 (41.0, 81.0)	46.0 (35.0, 66.0)	0.02
PSAd, ng/mL^2^	0.11 (0.07, 0.18)	0.12 (0.07, 0.16)	0.99
PI-RADS v2.1			< 0.001
2	72 (53%)	5 (12%)	
3	2 (1%)	16 (37%)	
4	25 (19%)	18 (42%)	
5	36 (27%)	4 (9%)	

*bpMRI* biparametric MRI, *DCE* dynamic contrast-enhanced, *DWI* diffusion-weighted imaging, *mpMRI* multiparametric MRI

For the 14 patients who underwent DCE due to significant artefact, the mean PI-QUAL v2 scores increased from 1.1 to 1.4 (*p* = 0.04) with the addition of DCE, upgrading four cases from inadequate to acceptable.

Compared to the non-monitored cohort, there was no significant difference in the prevalence of PI-RADS 3 scores, the rates of biopsy, or the detection of clinically significant or insignificant cancer (Table 3). There was also no significant difference in the diagnostic accuracy for detecting clinically significant cancer at a threshold score of PI-RADS ≥ 3 (Table 4).

**Table 3:** Differences in MRI and biopsy outcomes between on-table and no-monitoring groups

	On-table monitoring	No monitoring	Difference	*p*-value
	*N* = 178	*N* = 420		
PI-RADS v2.1 (%)			–	0.45
1	0 (0)	2 (< 1)		
2	77 (43)	204 (49)		
3	18 (10)	31 (7)		
4	43 (24)	85 (20)		
5	40 (22)	98 (23)		
Biopsy outcome (%)			–	0.87
No biopsy	84 (47)	212 (50)		
Benign	21 (12)	38 (9)		
ASAP	1 (1)	1 (< 1)		
3 + 3	14 (8)	40 (10)		
3 + 4	35 (20)	61 (15)		
3 + 5	1 (1)	5 (1)		
4 + 3	9 (5)	28 (7)		
4 + 4	3 (2)	7 (2)		
4 + 5	7 (4)	19 (5)		
5 + 3	0 (0)	2 (< 1)		
5 + 4	2 (1)	5 (1)		
5 + 5	1 (1)	1 (< 1)		
Biopsy rate, % (95% CI)	52.2 (44.9, 59.6)	49.5 (44.7, 54.3)	2.7 (−6.0, 11.5)	0.54
Significant cancer^a^, % (95% CI)	32.6 (25.7, 39.5)	30.7 (26.3, 35.1)	1.9 (−6.3, 10.0)	0.65
Insignificant cancer^b^, % (95% CI)	7.9 (3.9, 11.8)	9.5 (6.7,12.3)	−1.7 (−6.5, 3.2)	0.52

*ASAP* atypical small acinar proliferation, *CI* confidence interval, *PI-RADS v2.1* prostate imaging reporting & data system version 2.1

^a^ Clinically significant cancer is defined as greater or equal to Gleason grade 3 + 4

^b^ Insignificant cancer is defined as Gleason grade 3 + 3

**Table 4:** Diagnostic test accuracy between on-table and no-monitoring groups with 95% confidence intervals and statistical comparison using a two-sample *t*-test

	On-table monitoring, % (95% CI)	No monitoring, % (95% CI)	*p*-value
Sensitivity	98.3 (91.0, 99.7)	99.2 (95.8, 99.9)	0.56
Specificity	63.9 (54.9, 71.9)	70.7 (65.2, 75.6)	0.18
NPV	98.7 (93.0, 99.8)	99.5 (97.3, 99.9)	0.47
PPV	57.4 (47.7, 66.6)	60.8 (54.1, 67.2)	0.56

*CI* confidence interval, *NPV* negative predictive value, *PPV* positive predictive value

Assuming a tariff cost difference of £53 between MRI with and without contrast, £7,155 was saved during the 18-month period. Assuming the same rate of contrast use, extrapolating to the entire cohort across the same 18-month time period would have resulted in a saving of £56,677.

## Discussion

Our study compared on-table monitoring of bpMRI to standard-of-care default use of mpMRI. To our knowledge, this is the first study to prospectively adopt this approach, with the potential to maximise the advantages of both bpMRI and mpMRI approaches. On-table monitoring of prostate bpMRI in our cohort showed that 75.8% of patients avoided gadolinium contrast injection with comparable biopsy rates, reported PI-RADS 3 scores, insignificant cancer detection, and overall diagnostic accuracy for clinically significant disease compared to the standard non-monitored mpMRI cohort. This approach also offered substantial cost savings estimated at £4000 per 100 prostate MRIs or £38,000 per year at our centre [23].

The most common reason for requiring contrast injection was the presence of indeterminate lesions on bpMRI. According to PI-RADS v2.1, peripheral zone lesions scoring 3/5 on DWI require DCE for potential upgrading to 4/5. In contrast, lower or higher-scoring PZ lesions and all TZ lesions can be characterised using bpMRI alone [3]. Consequently, patients with larger, more conspicuous lesions or significant benign prostate hyperplasia can usually avoid gadolinium. This is reflected in the higher proportion of PI-RADS 2 and 5 scores in the monitored group who did not receive contrast, alongside significantly higher PSA levels and prostate volume in this group. Previous studies suggest that mpMRI may perform better in lower PSA (≤ 10 ng/mL) groups, whereas bpMRI has non-inferior performance in cohorts with a higher PSA level (> 10 ng/mL) [24], and in our cohort, only 5/43 (12%) patients with PSA > 10 ng/mL received gadolinium compared to 38/135 (28%) when PSA was ≤ 10 ng/mL.

DCE upgraded 13/27 patients from PZ PI-RADS 3 to 4, seven of whom had significant cancer. PI-RADS 4 lesions have a higher prevalence of significant cancer (59%) compared to PI-RADS 3 (16%) and are typically biopsied [25]. Practices for PI-RADS 3 lesions vary, and the impact of up-scoring lesions from 3 to 4 will depend on the approach of individual centres: those biopsying all PI-RADS 3 lesions may benefit less from DCE up-scoring than those that avoid biopsy in the subset of patients with lower PI-RADS score 3 and lower clinical risk based on PSAd. Among the 12 up-scored ‘PI-RADS 3 + 1’ cases, three had significant cancer despite PSAd < 0.15. Re-biopsy practices may also differ, as PI-RADS 4 lesions remain high-risk and may require repeat biopsy after negative results if sampling error is being considered [26–28]. Messina et al. found a lower prevalence of significant disease in ‘PI-RADS 3 + 1’ on mpMRI (14.5%) compared to PI-RADS 4 based on bpMRI (53.3%), recommending biopsy for ‘PI-RADS 3 + 1’ cases only if there is a high PSAd [29]. However, our study observed similar rates of significant cancer between these groups, including cases with lower PSAd, albeit in a smaller cohort.

The second most common reason for DCE was significant artefacts on bpMRI from pelvic metal work, patient motion, and rectal gas. Image quality is crucial for MRI diagnostic accuracy, and poor quality can lead to misclassification and indeterminate scoring, impacting the diagnostic pathway [30, 31]. PI-QUAL recommendations aid the assessment of mpMRI quality and its impact on diagnosing significant disease [32]. Hardware limitations may prevent some centres from meeting the PI-RADS minimal technical standards, [33, 34]. PI-QUAL v2 allows for scoring bpMRI and mpMRI and stipulates that good quality DCE imaging can upgrade the overall quality of the examination from inadequate to acceptable when both T2WI and DWI are deemed inadequate [22].

The reasons for significant artefacts in our monitored cohort were from patients rather than scanner or sequence factors. Pelvic metalwork, for example, is unavoidable and can have adverse effects on DWI particularly, but will be known prospectively, before scanning. Therefore, prior amendments can be made, such as using lower 1.5 T field strengths or spin-echo techniques [35, 36], and patients should receive gadolinium as standard. Unpredictable factors, such as movement or rectal gas, only become apparent during scanning, but may prospectively be mitigated by the use of antispasmodics and rectal preparation techniques [36–38]. DCE sequences are typically less prone to susceptibility artefacts than DWI and can therefore act as a ‘safety net’ for image quality, as well as lesion detection [35]. We observed an increase in mean PI-QUALv2 scores for those cases with significant artefacts with DCE reclassifying four cases from inadequate to being deemed acceptable. However, despite high-quality DCE, the majority of cases remained as PI-QUALv2 1/3 as “up-grading” still requires one of T2WI or DWI to be rated 4/4 for their individual sequence quality, further highlighting the importance of high-quality bpMRI. Even if categorical improvement in quality as judged by PI-QUALv2 is not achieved, the addition of DCE can still lead to improved reader confidence as MRI quality likely lies on a more nuanced spectrum than just three distinct categories. We also observed an increase in reader confidence in the monitored cohort with the addition of DCE, which was previously quoted as a benefit of DCE [39]. This may be attributed to helping classify indeterminate lesions or improving overall MRI quality, but even when DCE may not directly change PI-RADS score it may reduce uncertainty for centres using less objective Likert scoring systems.

The decision for on-table monitoring must be made during the scan, necessitating scheduling adjustments to accommodate the extra time for DCE sequence acquisition. Pre-determining the need for contrast during protocolling and scheduling would be advantageous. There are suggestions it may be beneficial to perform DCE routinely in patients with pelvic metalwork, lower PSA values or individuals where the risk of missing significant disease outweighs the risk of false positives and unnecessary biopsies [11]. Even if on-table monitoring would not increase scanning throughput, it allows for more personalised protocolling, reducing contrast-related side effects and costs. Another important consideration is how baseline bpMRI impacts treatment decisions for patients eventually diagnosed with cancer, for instance, DCE can be helpful for accurate staging, particularly seminal vesicle invasion [40]. Active surveillance with bpMRI may suffice if MRI quality is high, but prospective studies are needed to validate the safety of this approach with baseline bpMRI [6]. On-table monitoring by an experienced radiologist may not be practical for some centres due to staff availability and the time required for image review and decision-making and is a potential limitation to adoption. This setup may necessitate changes to MRI scheduling and staff rotas. Automated tools, such as artificial intelligence (AI), may offer a solution. Numerous studies have explored AI’s capability to evaluate scan quality, identify normal examinations, and perform PI-RADS classification, which could aid in decisions about contrast use [41, 42].

Our study has several limitations that should be acknowledged. First, the on-table monitoring cohort was collected prospectively during a regular weekly session, whereas the control cohort was collected throughout the week, potentially leading to a selection bias based on schedule. We used propensity score matching to help alleviate some of these potential biases, however, this reduced the sample size of our comparison group and is not a replacement for true randomisation. The follow-up for non-biopsied patients was a minimum of 12 months, and although a longer period would be optimal to identify potential false negative cases, this is commonly used in studies where immediate biopsy is not confirmed and allows for repeat PSA at 3–6 months and MRI if required, according to guidelines [1, 43–45]. Another limitation is that the study was conducted at a single large teaching hospital by expert radiologists and we did not assess potential inter-reader variability, which may limit the wider applicability of the results [19, 20]. Furthermore, PI-QUAL v2 scores were assigned only in retrospect to a specific subset of the cohort, as PI-QUAL v2 had yet to be published at the time of the study and PI-QUAL v1 cannot be applied to bpMRI. We did not collect data on the differences in scanning time between the two groups. The review process will likely take additional time but may be offset by not requiring DCE, and this should be recorded in future work. In this study, however, the appointment durations remained the same for both groups. The cost-saving estimates are simplified, and a complete cost-benefit analysis would need to consider additional factors such as the cost of consumables, the radiologist’s time to undertake the review and its impact on the resulting diagnostic pathway, including repeat imaging and biopsy rates.

In conclusion, on-table monitoring of prostate MRI could enable a significant proportion of patients to avoid gadolinium contrast and its associated side effects. This approach facilitates personalised protocolling, potentially maximising the diagnostic benefits of mpMRI with the cost savings of bpMRI.

## Supplementary information

Electronic Supplementary Material

## Abbreviations

ADC: Apparent diffusion coefficient
bpMRI: Biparametric MRI
DCE: Dynamic contrast-enhanced
DWI: Diffusion-weighted imaging
mp: Multiparametric
mpMRI: Multiparametric MRI
MRI: Magnetic resonance imaging
PCa: Prostate cancer
PI-RADSv2.1: Prostate imaging-reporting and data system v2.1
