# Supplementary material for: On-table monitoring of prostate MRI could enable tailored utilisation of gadolinium contrast
Source: Eur Radiol. 2025 Mar 15;35(9):5701–9. doi: 10.1007/s00330-025-11479-3 (PMC12350576; doi:10.1007/s00330-025-11479-3)
Supplement: Supplementary file 1 — Electronic Supplementary Material [file 330_2025_11479_MOESM1_ESM.pdf]

On-table monitoring of prostate MRI could enable tailored utilisation of gadolinium contrast

<u>Electronic Supplementary Material (ESM)</u>

Eur Radiol (2025) Syer T, Carmo B, Sanmugalingam N et al.

Supplementary Table 1. MR sequence technical specifications.

	**GE SIGNA Artist**	**GE Discovery MR750**	**SIGNA Premier**
Field Strength (T)	1.5	3.0	3.0
Receiver Coil	30AA+40PA	32Ch Cardiac	30AA+60PA
**T2WI axial**			
Scanning sequence	SE, PROP	SE	SE
MR acquisition type	2D	2D	2D
Field of view (mm^2)	180 - 210	180	180
Reconstructed matrix	512 x 512	512 x 512	512 x 512
Reconstructed resolution	0.35 x 0.35	0.35 x 0.35	0.35 x 0.35
Slice thickness (mm)	3.0	3.0	3.0
Slice gap (mm)	0.0	0.0	0.0
Repetition time (ms)	2606 - 9128	2500 - 5074	2401 - 3792
Echo time (ms)	79.5 - 87.8	96.8 – 107.1	96.6 – 164.3
Flip angle (º)	160	111	111
Number of averages	1	1.5	1.5
**T2WI sagittal**			
Scanning sequence	FSE, Cube	FSE, Cube	FSE, Cube
MR acquisition type	3D	3D	3D
Field of view (mm^2)	220	220	220
Reconstructed matrix	512 x 512	512 x 512	512 x 512
Reconstructed resolution	0.43 x 0.43	0.43 x 0.43	0.43 x 0.43
Slice thickness (mm)	2.0	2.0	2.0
Slice gap (mm)	0.0	0.0	0.0
Repetition time (ms)	3000	3002	3000
Echo time (ms)	87.8 – 118.9	113.3 - 121.0	81.2 – 100.7
Flip angle (º)	90	90	90
Number of averages	1	1	1
**DWI axial**			
Scanning sequence	EP	EP	EP
MR acquisition type	2D	2D	2D
Field of view (mm^2)	280	280	280
Reconstructed matrix	256 x 256	256 x 256	256 x 256
Reconstructed resolution	1.09 x 1.09	1.09 x 1.09	1.09 x 1.09
Slice thickness (mm)	3.0	3.0	3.0
Slice gap (mm)	0.0	0.0	0.0
Repetition time (ms)	4000 - 5963	3321 - 4566	3775 - 4803
Echo time (ms)	72.8 – 87.8	69.2 – 70.3	66.2 – 66.7
Flip angle (º)	90	90	90
Acquired b-values	100/550/1000	100/750/1400	100/750/1400
Synthetic b-values	1400/2000	2000/2500	2000/2500
Number of averages per b-value	4/8/12	2/6/6	2/6/6
**DCE axial**			
Scanning sequence	Fat -saturated GR	Fat -saturated GR	Fat -saturated GR
MR acquisition type	3D	3D	3D
Field of view (mm^2)	240	240	240
Reconstructed matrix	256 x 256	256 x 256	256 x 256
Reconstructed resolution	0.94 x 0.94	0.94 x 0.94	0.94 x 0.94
Slice thickness (mm)	3.0	3.0	3.0
Slice gap (mm)	0.0	0.0	0.0
Repetition time (ms)	6.16 – 6.85	4.08 – 4.68	4.01 – 4.20

Echo time (ms)	3.13	1.79 – 2.09	1.73 – 1.82
Flip angle (º)	13 - 15	13	13
Number of averages	0.7	0.7	0.7

(AA – Anterior Array, Ch – Channel, EP - Echoplanar, GE – General Electric, GR – Gradient Echo, MR – Magnetic Resonance, PA – Posterior Array, PROP – Propeller, SE – Spin Echo)

Supplementary Table 2. Reasons for readers to opt for additional DCE sequences and why DCE was helpful in patients who underwent DCE imaging in the on-table monitoring cohort.

	**Frequency (%)**
**Reason for DCE[a]**	n=43
Indeterminate	27 (63)
Significant artefact	14 (33)
DWI susceptibility	8 (19)
Motion	5 (12)
Total hip replacement	3 (7)
Diffuse change	3 (7)
**Reason DCE helpful**	n=43
Yes	29 (67)
Focal enhancement	13 (30)
Increased reader confidence	11 (26)
Features of inflammation	3 (7)
Appearance of asymmetrical CZ	2 (5)
No	14 (33)

[a] Some cases have more than one reason for requiring DCE
(CZ – Central Zone, DCE – Dynamic Contrast Enhanced Imaging, DWI – Diffusion-weighted Imaging)
